# The Impact of Surface Functionalization on the Biophysical Properties of Silver Nanoparticles

**DOI:** 10.3390/nano9070973

**Published:** 2019-07-03

**Authors:** Agnieszka Borowik, Kamila Butowska, Kinga Konkel, Rafał Banasiuk, Natalia Derewonko, Dariusz Wyrzykowski, Mykola Davydenko, Vsevolod Cherepanov, Viktor Styopkin, Yuriy Prylutskyy, Paweł Pohl, Aleksandra Krolicka, Jacek Piosik

**Affiliations:** 1 Laboratory of Biophysics, Intercollegiate Faculty of Biotechnology UG-MUG, University of Gdansk, 80-307 Gdansk, Poland; 2 Laboratory of Biologically Active Compounds, IFB UG-MUG, University of Gdansk, 80-307 Gdansk, Poland; 3 Laboratory of Virus Molecular Biology, IFB UG-MUG, University of Gdansk, 80-307 Gdansk, Poland; 4 Faculty of Chemistry, University of Gdansk, 80-308 Gdansk, Poland; 5 Department of Biophysics and Medical Informatics, Taras Shevchenko National University of Kyiv, 01601 Kyiv, Ukraine; 6 Institute of Physics of NAS of Ukraine, 03028 Kyiv, Ukraine; 7 Faculty of Chemistry, Division of Analytic Chemistry and Chemical Metallurgy, Wroclaw University of Technology, 50-373 Wroclaw, Poland

**Keywords:** silver nanoparticles, synthesis, surface functionalization, mutagenicity, Ames test, toxicity

## Abstract

Among metal-based nanoparticles, silver nanoparticles (AgNPs) are particularly appealing because of their stability, functionality, and documented antimicrobial properties. AgNPs also offer the possibility of different surface modifications. In this work, we functionalized AgNPs with thiobarbituric acid or 11-mercaptoundecanoic acid residues to improve the nanoparticles’ biological activities. Subsequently, we assessed the physicochemical properties of newly synthesized AgNPs using a wide range of biophysical methodologies, including UV/vis and fluorescence spectroscopy, atomic force and scanning electron microscopy, and dynamic light scattering and isothermal titration calorimetry. Next, we examined the effect of nanoparticles functionalization on AgNPs mutagenicity and toxicity. Our study revealed that AgNPs’ surface modification affects nanoparticles aggregation, and also impacts nanoparticles’ interaction with model acridine mutagen ICR-191. AgNPs coated with MUA showed the most interesting interactions with tested ICR-191, slightly modulating its toxicity properties by decreasing the viability in treated cells.

## 1. Introduction

Silver has been present in human beings’ daily lives and can be used, widely, in its different states: metallic, soluble, and insoluble. Silver nanoparticles (AgNPs), when suspended in a solvent, are referred to as colloidal silver [1,2,3,4,5,6]. Despite their interesting properties, AgNPs nanoparticles play an important role as templates for further surface modifications. Such surface modifications can endow AgNPs with novel functionalities, and thus further extend the possibilities of their broad application. For instance, the addition of monosaccharides residues on nanoparticles can modulate AgNPs’ cellular uptake and toxicity [7,8].

AgNPs penetrate into cells through passive diffusion or via the endocytosis pathway. After entering the cell, AgNPs may interact with macromolecules, lose their surface coating agents, or aggregate in biological media. Because of particles’ surface oxidation, Ag^+^ ions are being continuously released. Any such processes may affect the silver’s toxicity towards the cell [4,5,7,8,9,10]. It should be highlighted that both AgNPs and Ag^+^ ions might interfere with multiple components of cells. AgNPs are able to non-covalently interact with different proteins, and interaction mechanisms may be affected by particle size, surface coating, and silver concentration [3,7,8,10,11]. One of the proposed scenarios assumes the existence of electrostatic interactions between silver ions on the nanoparticles’ surface and electron donor groups of proteins, such as tiols, or metal chelation sites within enzymes [3]. Silver ion release connected with AgNPs’ surface oxidation enhances the generation of reactive oxygen species (ROS) in cells. This, in turn, together with mitochondrial membrane disruption, disturbs the respiratory chain. In addition, AgNPs can inhibit the DNA replication process by direct and indirect interference with nucleic acids’ bases, which results in a local DNA condensation [3]. Moreover, AgNPs might deactivate signaling pathways, induce G1 arrest, and block cells in the S-phase, which inhibits cell proliferation and leads to apoptosis activation [10,11,12].

Extensive usage of AgNPs leads to increased human exposure and potential environmental contamination. AgNPs might exert noticeable toxicity against tested cell eukaryotic lines and laboratory animals (like rodents or aquatic organisms) [3,4,7]. McShan et al. [10] suggest that it is very difficult to predict which part of the observed toxicity effects is caused by an ionic form of silver (Ag^+^), and which part is rather related to its nano form. It was already confirmed that large doses of silver or its compounds might be accumulated in diverse body tissues, leading to argyria, resulting in characteristic blue skin pigmentation [3,10,13]. In addition, despite the increasing amount of toxicity studies, available AgNPs’ toxicity assessment still remains inadequate and inconclusive [2,5,14]. Unfortunately, there is still a limited knowledge about the possible relationship between physicochemical properties of nanoparticles and observed biological effects, induced by AgNPs’ presence. The type and strength of these alterations are closely connected with AgNPs’ size, concentration, presence of chemical coating, animal species, and route of exposure. This leads to the hypothesis that it is almost impossible to designate one universal mechanism responsible for AgNPs-related biological changes. This is why a rigorous physicochemical and biological characterization of novel AgNPs is highly recommended. 

In the present article, we propose a new method of AgNPs’ surface modification, allowing obtaining nanostructures with attached thiobarbituric acid (hereafter called AgNP-TBA) or 11-mercaptoundecanoic acid (hereafter called AgNP-MUA) residues. Such modification might result in the improvement of AgNPs’ biological activities. Subsequently, we performed AgNPs’ full physicochemical characterization, as well as evaluated their potential to directly interact with biologically active substances. To achieve this, we examined a model acridine mutagen ICR-191 with well-known biological and biophysical properties. ICR-191 is already described by our research group as an active compound able to interact with nanoparticles such as C_60_ fullerene [15] or nanoplatinum [16]. Further, we checked if introduced coating agents have changed the mutagenicity and toxicity of newly synthesized particles, comparing to uncoated AgNPs. We also evaluated whether AgNPs, AgNP-TBA and AgNP-MUA can modulate ICR-191 activity.

## 2. Materials and Methods 

### 2.1. Materials

Model acridine mutagen ICR-191 was purchased from Sigma Aldrich Chemical Company (Steinheim, Germany). Its stock solution (concentration 10^−3^ M) was obtained by dissolving the weight amount in distilled water. Concentrations of ICR-191 solutions were determined spectrophotometrically, using molar absorption coefficients ε_421.5_ = 7.5624×10^3^ M^−1^ cm^−1^.

*Salmonella typhimurium* TA98 strain used in the mutagenicity Ames test was purchased from Trinova Biochem GmbH, Giessen, Germany. Nutrient broth media, ampicillin, biotin, and histidine used in the Ames test were purchased from Sigma Aldrich Chemical Company (Steinheim, Germany).

In proliferation assays, the human keratinocyte cell line (HaCaT) and the human melanoma cell line (MelJuSo) were employed. HaCat cells were obtained from the Department of Microbiology, Tumor, and Cell Biology, Karolinska Institute (Stockholm, Sweden), while the MelJuSo cells were from the Department of Medicinal Microbiology, Leiden University Medical Center (Leiden, The Netherlands). The Dulbecco’s modified Eagle’s medium (DMEM), bovine serum, L-glutamine, glucose, penicillin, and streptomycin used in the cell lines experiments were purchased from Sigma Aldrich Chemical Company (Steinheim, Germany).

### 2.2. Nanoparticle Synthesis and Derivatization

The nanoparticle synthesis was performed as described in Banasiuk et al. [17]. To a borosilicate bottle, containing 100 mL of deionized water, 200 mg polyvinylpyrrolidone (Sigma Aldrich Chemical Company, Steinheim, Germany), 0.5 mL 5.25% sodium hypochlorite, and silver nitrate to the final concentration of 4 mM were added. After combining the reagents, the mixture was irradiated using a 5 W blue LED lamp (λ_max_ = 420 nm) for 10 min. The purple AgNPs suspension was concentrated ten times using a centrifuge (15,000 RCF, 15 min). Subsequently, 45 mL of concentrated AgNPs was put into a temperature controlled mechanical shaker set to 70 °C for further surface modifications. After preheating, 2 mg/mL of thiobarbituric acid (TBA, Sigma Aldrich Chemical Company, Steinheim, Germany) or 11-mercaptoundecanoic acid (MUA, Sigma Aldrich Chemical Company, Steinheim, Germany) was added. The mixture was kept at 70 °C for 4 h, after which it was kept at 20 °C for another 72 h. After the incubation period, nanoparticles were collected, and the sediment discarded. As a result of the described procedures, three different types of nanoparticles were synthesized: naked AgNPs, AgNPs with attached thiobarbituric acid residues (called AgNP-TBA), and AgNPs with attached 11-mercaptoundecanoic acid residues (called AgNP-MUA).

### 2.3. Synthesis Efficacy Determination

To separate the synthesized AgNPs from unreacted silver ions and the remains of unattached TBA and MUA residues, the suspensions were filtered through 0.2 μm mixed cellulose–ester syringe filters and then concentrated by the centrifugation (15,000 rcf, 30 min). The supernatants were discarded and the resulting sediments were suspended in 2 mL of deionized water. To determine the efficacy of silver nanoparticles’ synthesis, the samples of the separated AgNPs, AgNP-TBA, and AgNP-MUA were digested and the concentration of silver was determined. Prior to analysis, the samples were mixed using a vortex for 1 min. Then, 50 µL was taken and introduced to 10 mL PP vials and treated with 500 µL of a concentrated HNO_3_ solution (ACS grade, 70%, Sigma-Aldrich, Steinheim, Germany). Afterwards, the vials with the samples and the oxidizing reagent were put into a water bath (temp. ~105 °C) and kept therein for 60 min to completely digest the AgNPs and the organic matrices associated with them. After that, the resulting aliquots were cooled down and reconstituted with water to obtain a final volume of 50 mL. The concentrations of Ag in the resultant sample solutions were established using an Agilent inductively coupled plasma optical emission spectrometry (ICP OES) instrument, model 720 with an OneNeb nebulizer and a cyclonic spray chamber for pneumatic nebulization of the standards solutions and tested samples. The quantification of silver was carried out by two standard additions at the level of 1 and 2 µg/mL. Considering the masses of the samples and the final volumes of the sample solutions achieved, the established amounts of Ag were interpreted as the AgNPs concentration in each sample. Obtained data were presented as average values ± relative standard deviation (RSD), which was calculated by multiplying the standard deviation value by 100 and dividing this product by the average. 

### 2.4. Scanning Electron Microscopy (SEM)

To visualize the shape and properties of newly synthesized AgNPs (naked AgNPs, AgNP-TBA, and AgNP-MUA), the scanning electron microscopy (SEM) technique was used. Tested samples were deposited on cleaned silicon p-type (100) plates. Then, images of deposits were obtained in the secondary electron mode at 25 kV energy using a JSM-35 scanning electron microscope (JEOL, 3-1-2 Musashino, Akishima, Tokyo, Japan). AgNPs were studied in the as-prepared pristine state and, sometimes, images of large particles were slightly distorted as a result of their charging under electron beam irradiation.

### 2.5. Atomic Force Microscopy (AFM)

Atomic force microscopy (AFM) studies were performed to determine the size and surface morphology of synthesized AgNPs on a "Solver Pro" microscope (NT-MDT Spectrum Instruments, Moscow, Russia), equipped with an optical microscope. Measurements were carried out in tapping mode using AFM probes RTESPA (Bruker, Mannheim, Germany, 300 kHz, 40 N/m). A freshly cleaved atomically smooth surface of mica (V1 grade, SPI Supplies) was used as a substrate. A droplet of suspension (0.2 µL) containing nanoparticles (naked AgNPs, AgNP-TBA, and AgNP-MUA) was applied onto a limited area of the mica and left until complete evaporation of the water. The optimal measurement concentrations of AgNPs were achieved by a dilution of stock solutions with distilled water.

### 2.6. Nanoparticles Hydrodynamic Size and Electrokinetic Potential Measurements

We used the dynamic light scattering (DLS) and zeta potential measurements (ζ) for ascertaining the hydrodynamic size and electrokinetic potential of newly synthesized AgNPs: naked AgNPs, AgNP-TBA, and AgNP-MUA. All solutions were analysed in their original concentrations obtained after synthesis (2.65 × 10^3^ µg/mL, 625 µg/mL, and 65.5 µg/mL, respectively). In the second ζ potential measurement, the concentrated solutions were diluted in distilled water. The tested volumes of nanoparticles were 1.5 mL. Measurements were conducted on Zetasizer Nano-ZS90 (Malvern, Malvern, Worcestershire, UK) at 25 °C in triplicate. The results were evaluated using the Smoluchowski approximation, which is known to be rigorously valid only for spherical-like particles.

### 2.7. UV-Vis Spectrophotometric Measurements

Light absorption spectra were measured in a wide wavelength range of 300–800 nm with 0.5 nm intervals, using Analytik Jena, Jena, Germany, Specord 50 Plus spectrophotometer with temperature stabilized by Peltier thermostat (25 ± 0.1 °C). Measurements were done in quartz cuvettes (1 cm light path) containing 2 mL 0.2 M sodium-phosphate buffer, pH 6.8. In the first experiment, absorbance spectra of AgNPs (naked AgNPs, AgNP-TBA, or AgNP-MUA) per se were determined (concentration ranges: 6.61–83.41 µg/mL, 1.56–22.59 µg/mL, and 0.16–7.66 µg/mL, respectively). In the second approach, naked AgNPs, AgNP-TBA, or AgNP-MUA nanoparticles (primary concentration: 64.63 µg/mL, 19.67 µg/mL, and 6.49 µg/mL, respectively) were titrated with an increasing amount of ICR-191 (concentration ranges: 25.19–97.92 µM, 25.01–97.23 µM, and 23.28–90.68 µM, respectively). To minimize the impact of strong AgNPs’ homoaggregation, previously measured absorbance spectra of tested nanoparticles titrated with distilled water (added in the same volumes as tested mutagen) were subtracted from the newly registered data. Resulting data were normalized to ICR-191 concentration and presented as molar extinction coefficient spectra. 

### 2.8. Fluorescence Spectroscopy

The ICR-191 fluorescence emission spectrum (excitation wavelength = 340 nm, emission wavelengths = 400–650 nm) was measured and the maximum fluorescence peak at 496 nm was observed (data not shown). Afterwards, the intensity of the fluorescence spectra was determined for mixtures of ICR-191 (primary concentration: 119.73 µM) titrated with increasing amounts of AgNPs: naked AgNPs, AgNP-TBA, or AgNP-MUA (concentration ranges: 25.30–98.38 µg/mL, 5.97–23.20 µg/mL, and 0.63–2.43 µg/mL, respectively). All samples were measured in quartz cuvettes at 25 °C, using FP-8500 Spectrofluorimeter (Jasco, Easton, MD, USA). Obtained data are expressed as the mean relative fluorescence unit (RFU).

### 2.9. Silver Nanoparticles Aggregation with ICR-191

The analysis of the aggregates size distributions was conducted by the DLS technique for naked AgNPs, AgNP-TBA, and AgNP-MUA nanoparticles alone (64.63 µg/mL, 15.24 µg/mL, and 3.12 µg/mL, respectively) and mixed with ICR-191 (final mutagen concentrations: 78.87 µM and 77.03 µM, respectively). Using Zetasizer Nano ZS (Malvern, Worcestershire, UK), the intensity of the scattered light was measured at 25 °C with a He–Ne laser (633 nm, 4 mW), at a scattering angle of 173°. The tested suspensions were placed in polystyrene cuvettes. The results were evaluated using the Smoluchowski approximation, which is known to be rigorously valid only for spherical-like particles. All measurements were performed in triplicate. Obtained data are shown as size distributions (nm) of light scattering particles (in accordance to their hydrodynamic diameters) by intensity (%).

### 2.10. Thermodynamical Properties of Silver Nanoparticles and ICR-191 Aggregation

Experiment1s describing the thermodynamical properties of mutagen–anoparticles aggregation were performed as described before [18] using an AutoITC isothermal titration calorimeter (ITC) (MicroCal Inc. GE Healthcare, Northampton, MA, USA) in water at 25 °C. The volume of both sample and reference cells (containing distilled water) was 1.4491 mL. Three independent experiments performed for naked AgNPs, AgNP-TBA, as well as AgNP-MUA consisted of multiple injections of 10 µL ICR-191 portions (initial concentration 295.5 µM) to the sample cell containing tested AgNPs’ suspension (initial concentrations were experimentally determined, Table 1), followed by the measurements of the heat of the process as a function of time. An initial 2 µL injection was discarded from each data set in order to remove the effect of titrant diffusion across the syringe tip during the equilibration process. Each injection lasted 20 s. In order to achieve homogeneous mixing in the cell, the stirrer speed was kept constant at 300 rpm. Background titrations were measured for ICR-191 solution titrated with water and water as a titrant for AgNPs. The registered results of background titrations were then subtracted from final experimental results to account for the heat of dilution. All suspensions were degassed before titrations were performed. The titrant was injected at 4 min intervals to ensure that the titration peak returned to baseline prior to the next injection. The obtained data, specifically the heat normalized per mole of injectant, were processed with Origin ver. 7.0 from MicroCal (MicroCal, Northampton, MA, USA).

### 2.11. Bacterial Reverse Mutation Assay—Ames Test

To examine the potential mutagenic properties of newly synthesized naked AgNPs, AgNP-TBA, and AgNP-MUA nanoparticles, bacterial reverse mutagenicity test (the Ames test) was performed with *Salmonella typhimurium* TA98 strain, without metabolic activation, in accordance with the procedure described by Mortelmans and Zeiger [18], with further modifications applied by Golunski et al. [19]. Briefly, a mixture containing 100 µL of overnight culture of *S. typhimurium* TA98 (corresponding to 1 × 10^8^ colony forming units), 50 µL of 3% NaCl, and 100 µL of tested nanoparticles (or sterile water for the negative control) was incubated for 4 h in darkness at 37 °C and 220 rpm. Subsequently, the mixture was centrifuged, and the bacterial pellet was washed with 0.75% NaCl and resuspended in 300 µL of 0.75% NaCl solution containing 0.1 µmol histidine and 0.1 µmol biotin. Finally, bacterial suspension was spread on a glucose minimal (GM) plate. After 48 h incubation at 37 °C in darkness, the number of revertant colonies was calculated. All experiments were performed in triplicate. Optimal mutagen concentration (0.2 mg/plate) used as a positive control was chosen after testing ICR-191 mutagenic activity in a broad concentration range (data not shown). AgNPs concentrations tested in this assay were not lethal towards *S. typhimurium*. Their ranges’ selection was carefully preceded by minimum inhibitory concentrations (MIC) and minimum bactericidal concentrations (MBC) determination for used bacteria, in accordance with the procedure described by Krychowiak et al. [20]. Probable toxicity towards bacteria was controlled by observation of the auxotrophic background (background lawn).

### 2.12. AlamarBlue Cell Viability Assay

HaCaT and MelJuSo cells were seed on a 96-well plate (2 × 10^4^/well) and incubated in a humidified atmosphere containing 5% CO_2_, at 37 °C, overnight. Then, cells were treated with 45.7 µM ICR-191, 0.08 and 0.008 µg/mL naked AgNPs, 0.08 and 0.008 µg/mL AgNP-TBA, 0.065 and 0.0065 µg/mL AgNP-MUA, or a mixture of AgNPs and ICR-191 (final volume 90 µL/well) in three replicates, and incubated for 24 or 48 h. The additional control wells consisted only of untreated cells in the culture medium. Subsequently, 10 µL of AlamarBlue (Bio-Rad, Hercules, CA, USA) was added into each well and further incubated for the next 4 h, in humidified atmosphere containing 5% CO_2_, at 37 °C. Finally, absorbance was measured at wavelengths of 570 and 600 nm. Pure medium was used as blank. Percentage of the AlamarBlue reduction was calculated as the difference between treated and control cells, according to the protocol provided by the manufacturer.

## 3. Results

### 3.1. Synthesis Efficacy Evaluation

The efficiency of the synthesis and surface functionalization was determined by the final amount of obtained nanoparticles: naked AgNPs, AgNP-TBA, and AgNP-MUA. The number of synthesized nanostructures was interpreted as the total concentration of silver in every sample. Measured silver concentrations for each type of nanoparticles stock solution are presented in Table 1. It can be noticed that highest silver concentration was established for naked AgNPs (2650 µg/mL). The solution of AgNPs functionalized with TBA contains 625 µg/mL of silver, while the AgNP-MUA sample is ten-fold less concentrated (62.5 µg/mL). These determined concentrations were used in further experiments. 

### 3.2. Physicochemical Characterization of Silver Nanoparticles

Particles’ size, shape, and distributions in newly synthesized colloidal silver solutions were determined by several techniques, such as SEM, AFM, DLS, and UV/vis spectroscopy. 

**Figure 1:**
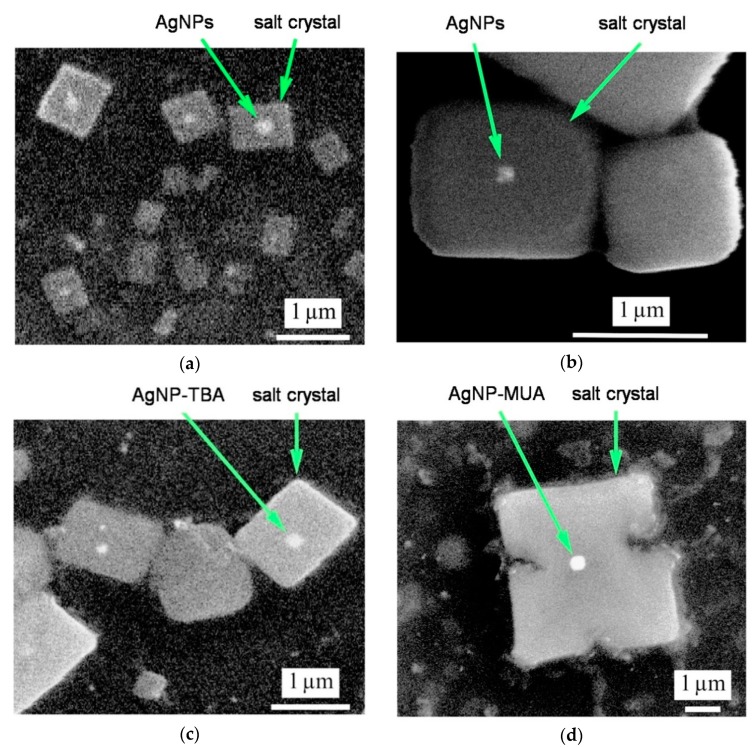
Scanning electron microscopy (SEM) micrographs of synthesized silver nanoparticles (AgNPs): (**a**) SEM micrograph of naked AgNPs. Arrows indicate AgNPs and salt crystals; (**b**) Magnified zone of naked AgNPs showing the salt crystal; (**c**) Micrograph showing of AgNP-thiobarbituric acid (TBA) nanoparticles and concomitant salt crystals; (**d**) Magnified view of AgNP-11-mercaptoundecanoic acid (MUA) nanoparticles within a salt crystal.

In every SEM image presented in Figure 1, there are crystalline objects of a rectangular or rhombic shape with the linear dimensions ≥1 μm. These objects were identified as salt crystals by their characteristic shape and ability to dissolve in water. To verify their nature, we applied measurements of the backscattered electrons mode, where the registered signal level depends on the average atomic number. The signal from the bright spots visible inside the salt crystals was much stronger than the average signal over the crystal, indicating that spots correspond to synthesized AgNPs. AgNPs thus acted as nucleation centers for salt crystallization during the evaporation of water from a drop of deposited mixture. A magnified SEM image of naked AgNPs is presented in Figure 1b, AgNP-TBA in Figure 1c, and AgNP-MUA in Figure 1d. All three forms of AgNPs resemble a square-like shape.

**Figure 2:**
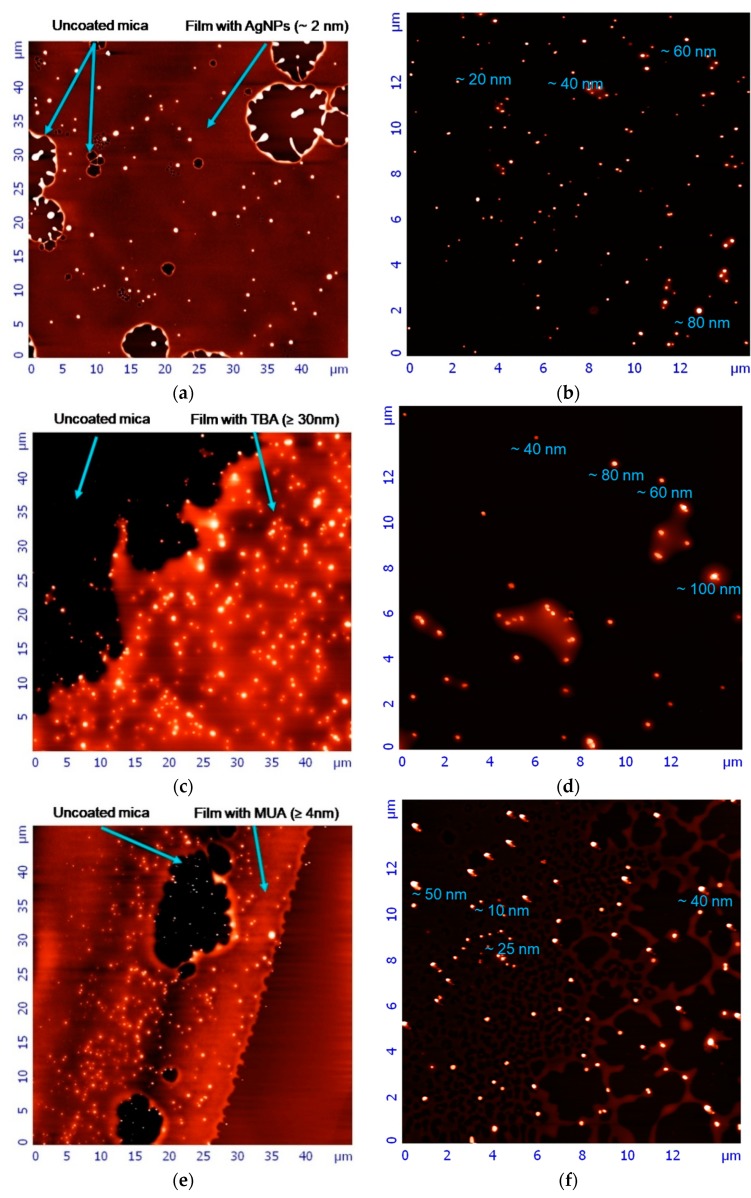
Atomic force microscopy (AFM) images of newly synthesized AgNPs. (**a**) Images of naked AgNPs precipitated on a mica after 100-fold dilution. Arrows indicate the uncoated mica and the polymer layer height; (**b**) images of naked AgNPs precipitated on the uncoated mica after 100-fold dilution; (**c**) images of AgNP-TBA nanoparticles precipitated on a mica from the original solution. Arrows indicate the uncoated mica and the polymer layer height; (**d**) images of AgNP-TBA nanoparticles precipitated on the uncoated mica from the original solution; (**e**) images of AgNP-MUA nanoparticles precipitated on a mica after 30-fold dilution. Arrows indicate the uncoated mica and the polymer layer height; (**f**) images of AgNP-MUA nanoparticles precipitated on the uncoated mica after 30-fold dilution. Nanoparticles’ heights are given below measured individuals.

The AFM visualization of AgNPs was hampered by a layer of polyvinylpyrrolidone polymer (residue after synthesis), which formed on the mica surface after evaporation of the water. In Figure 2, a polymer film containing the three different classes of AgNPs is shown, as well as areas of uncoated mica. To reduce the impeding effect of the polymer film, we diluted the stock solutions of tested nanoparticles with distilled water prior to the analysis. This allowed us to determine the average height of synthesized nanostructures. The height of the polymer layer (~2 nm) present in naked AgNP samples after 100-fold dilution of stock solution is marked in Figure 2a. In Figure 2b, naked AgNP particles’ size oscillated within the 15–80 nm range, with the main size maintaining at 40–60 nm. In Figure 2c, there is a non-diluted polymer layer in the AgNP-TBA sample ≥30 nm. The AgNP-TBA particles’ height, shown in Figure 2d, were within the 40–100 nm range, with the main size of 50–80 nm. For comparison, in Figure 2e, where the polymer layer was diluted 30-fold, the AgNP-MUA sample was ≥4 nm. The smallest AgNP-MUA, presented in Figure 2f, was within 1–50 nm in size, with a main size of 25–40 nm. On the basis of AFM data, we estimated the ratio of particle concentrations in suspensions. We also observed that naked AgNPs’ suspension had noticeably 2–3 times more particles than AgNP-TBA suspension, and the least rich in particles solution was AgNP-MUA suspension, which had a smaller amount of nanoparticles.

The aggregation pattern of synthesized AgNPs was examined with the DLS technique. Analyzed particles hydrodynamic diameters and electrokinetic potential (ζ potential) values are summarized in Table 2. In the aqueous suspension, naked AgNPs had a size of 110 nm. When silver particles were coated with TBA and MUA residues, their sizes increased to 273 nm and 199 nm, respectively. All of the tested AgNPs demonstrated a slightly negative average surface charge and the lowest one was observed for AgNP-TBA (−7.92 mV). Interestingly, after a 10-fold dilution of nanoparticles original stock suspensions with distilled water, the measured ζ potential value in each case was reduced to the level of approximately −13 mV. The particle size distribution can be also derived from the polydispersity index (PDI) value. The registered PDI values in the range of 0.2–0.3 indicate a moderately disperse distribution of the tested samples (Table 2).

**Figure 3:**
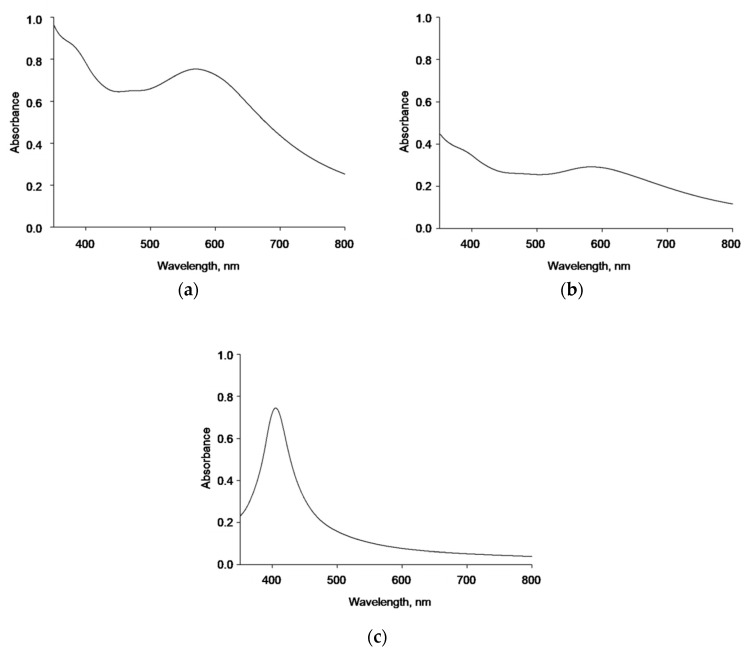
Spectrophotometric analysis of AgNPs in 0.2 M sodium-phosphate buffer (pH = 6.8). (**a**) Absorbance spectrum of 83.41 µg/mL naked AgNPs; (**b**) absorbance spectrum of 22.59 µg/mL AgNP-TBA nanoparticles; (**c**) absorbance spectrum of 7.66 µg/mL AgNP-MUA nanoparticles.

Further characterization of synthesized AgNPs was made by UV/vis spectrophotometric analysis. The absorbance spectra of the tested samples registered in the range of 350–800 nm are shown in Figure 3. The naked AgNPs in sodium-phosphate buffer had an absorbance spectra peak at about 570 nm (Figure 3a). The subtle peak at 585 nm, shifted to the red, might be observed in the AgNP-TBA suspension (Figure 3b). On the contrary, a strong peak at 405 nm was measured for AgNP-MUA particles, which is shifted to the blue when compared with the naked AgNPs and AgNP-TBA suspensions (Figure 3c). Moreover, all three types of AgNPs strongly scatter the light, which is probably because of their homoaggregation processes.

### 3.3. Identification of Potential Interactions between Silver Nanoparticles and ICR-191 

**Figure 4:**
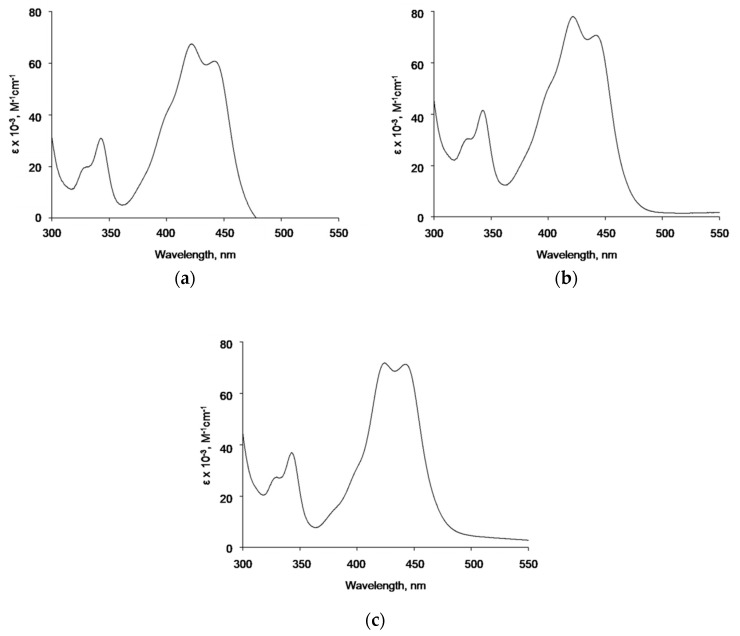
UV/vis spectrum of AgNPs titrated with ICR-191 reduced by AgNPs’ absorbance. (**a**) Spectrum of naked AgNPs (initial concentration 64.63 µg/mL) titrated with ICR-191 (concentration range 25.19–97.92 µM); (**b**) spectrum of AgNP-TBA (initial concentration 19.67 µg/mL) titrated with ICR-191 (concentration range 25.01–97.23 µM); (**c**) spectrum of AgNP-MUA (initial concentration 6.45 µg/mL) titrated with ICR-191 (concentration range 23.28–90.68 µM).

UV/vis spectroscopy was used to assess a possible interaction between model acridine mutagen ICR-191 and different types of AgNPs. Knowing that tested AgNPs tend to form homoaggregates, we performed two-step spectrophotometric experiments. First, naked AgNPs, AgNP-TBA, or AgNP-MUA were titrated with an increasing amount of ICR-191 (concentration ranges: 25.19–97.92 µM, 25.01–97.23 µM, and 23.28–90.68 µM, respectively) and absorbance spectra were registered in the range of 300–800 nm. Next, to minimize the negative impact of strong AgNPs homoaggregation, we measured absorbance spectra of naked AgNPs, AgNP-TBA, or AgNP-MUA titrated with distilled water added in the same volumes as the tested mutagen. Lastly, spectra obtained in both steps were subtracted from each other, and so resulting data were normalized to ICR-191 concentration and are presented as molar extinction coefficient spectrum in Figure 4a for naked AgNPs, Figure 4b for AgNP-TBA, and Figure 4c for AgNP-MUA. Discrete batho- and hypochromic shifts were observed for naked AgNPs and AgNPs coated with MUA, indicating the probability of direct interactions between those nanoparticles and ICR-191. Such spectral changes might suggest that at least two forms of ICR-191 exist in tested mixtures: free and non-covalently bounded with nanoparticles.

**Figure 5:**
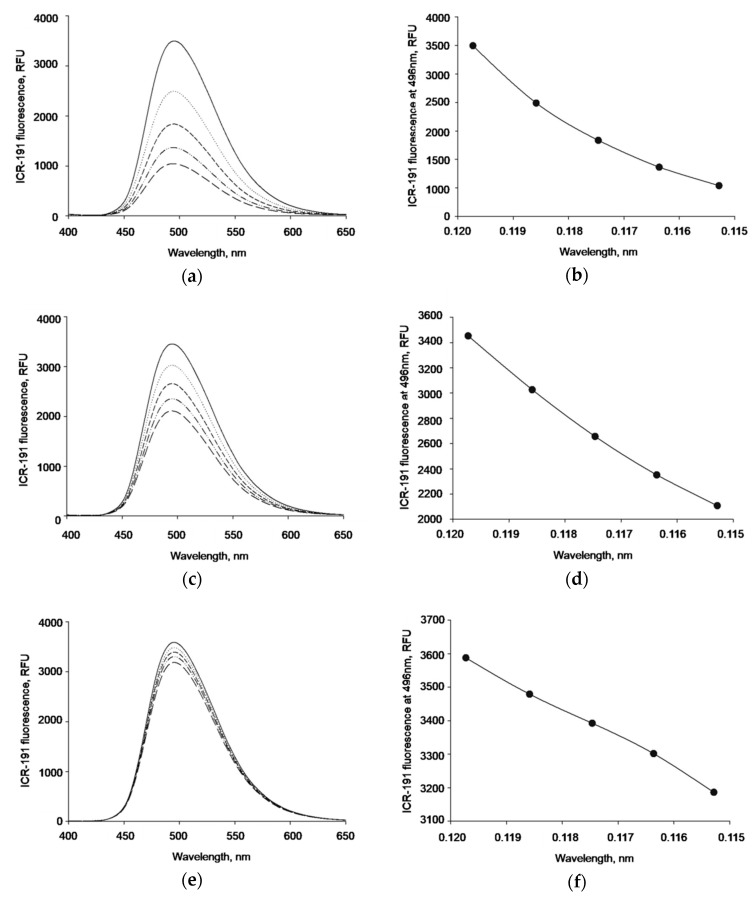
ICR-191 fluorescence changes caused by AgNPs. (**a**) Fluorescence emission spectrum of ICR-191 (primary concentration: 119.73 µM) titrated with increasing amount of naked AgNPs (concentration range: 25.30–98.38 µg/mL); (**b**) data registered for naked AgNPs shown as a relationship between the fluorescence intensity at the maximum point (496 nm) and the ICR-191 concentration; (**c**) fluorescence emission spectrum of ICR-191 (primary concentration: 119.73 µM) titrated with increasing amount of AgNP-TBA (concentration range: 5.97–23.20 µg/mL); (**d**) data registered for AgNP-TBA shown as a relationship between the fluorescence intensity at the maximum point (496 nm) and the ICR-191 concentration; (**e**) fluorescence emission spectrum of ICR-191 (primary concentration: 119.73 µM) titrated with increasing amount of AgNP-MUA (concentration range: 0.63–2.43 µg/mL); (**f**) data registered for AgNP-MUA shown as a relationship between the fluorescence intensity at the maximum point (496 nm) and the ICR-191 concentration. RFU—relative fluorescence unit.

In the next experiment, the ability of acridine mutagen to emit fluorescence was used to examine potential interactions between ICR-191 and synthesized AgNPs. We assumed that the formation of heteroaggregates between AgNPs and ICR-191 should result in changes in the intensity of fluorescence. The fluorescence intensities measured for mixtures of ICR-191 (primary concentration: 119.73 µM) titrated with increasing amounts of naked AgNPs (concentration range: 25.30–98.38 µg/mL) are presented in Figure 5a, while Figure 5c presents spectra of ICR-191 titrated with AgNP-TBA (concentration range: 5.97–23.20 µg/mL) and Figure 5e presents spectra of ICR-191 titrated with AgNP-MUA (concentration range: 0.63–2.43 µg/mL). Interestingly, the visible decrease in fluorescence intensity at the maximum point of 496 nm in Figure 5a,b supports the fact that naked AgNPs have the highest aggregation rate with ICR-191 molecules, as the resulting light emission properties are affected the most.

**Figure 6:**
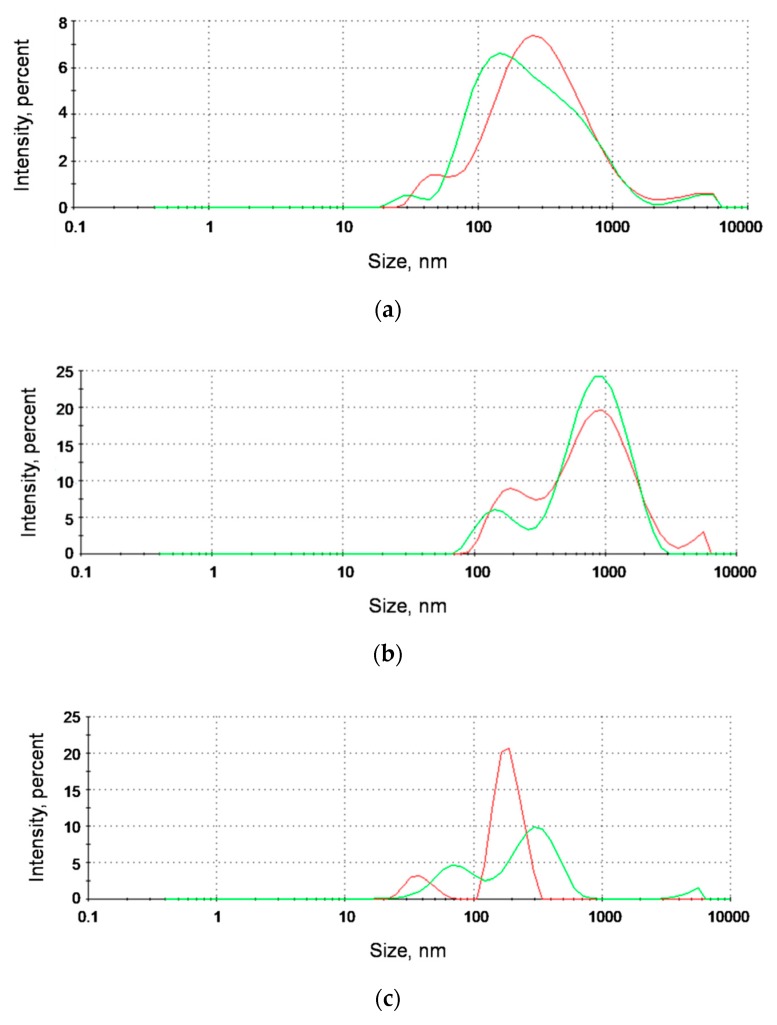
Mixed aggregation of AgNPs with ICR-191. (**a**) Hydrodynamic diameter of 64.63 µg/mL naked AgNPs marked in red equals 371.9 nm. Overall average size of naked AgNPs–ICR-191 aggregates marked in green (concentrations: 64.63 µg/mL and 78.87 µM, respectively) equals 322.2 nm; (**b**) hydrodynamic diameter of 15.24 µg/mL AgNP-TBA nanoparticles marked in red equals 199.3 and 996.9 nm. Overall average size of AgNP-TBA–ICR-191 aggregates marked in green (concentrations: 15.24 µg/mL and 78.87 µM, respectively) equals 158.6 and 954.8 nm; (**c**) hydrodynamic diameter of 3.12 µg/mL AgNP-MUA nanoparticles marked in red equals 38.9 and 189.8 nm. Overall average size of AgNP-MUA–ICR-191 aggregates marked in green (concentrations: 3.12 µg/mL and 77.03 µM, respectively) equals to 73.8 and 308.0 nm. Measurements were performed in triplicate, and data are shown as average values.

For further confirmation, another DLS assay was performed. We determined hydrodynamic diameters of analyzed nanoparticles in the presence of ICR-191. The results measured for naked AgNPs are shown in Figure 6a, for AgNP-TBA in Figure 6b, and for AgNP-MUA in Figure 6c. Surprisingly, in Figure 6c, we observed an extension in the average size of mixed aggregates containing AgNP-MUA and ICR-191 (concentrations: 3.12 µg/mL and 77.03 µM, respectively). AgNP-MUA particles themselves had sizes equal to 38.9 and 189.8 nm, and after the addition of ICR-191, their diameter increased to 73.8 and 308.0 nm. This enlargement in the hydrodynamic radius size was likely the result of the formation of AgNP-MUA–ICR-191 heteroaggregates.

**Figure 7:**
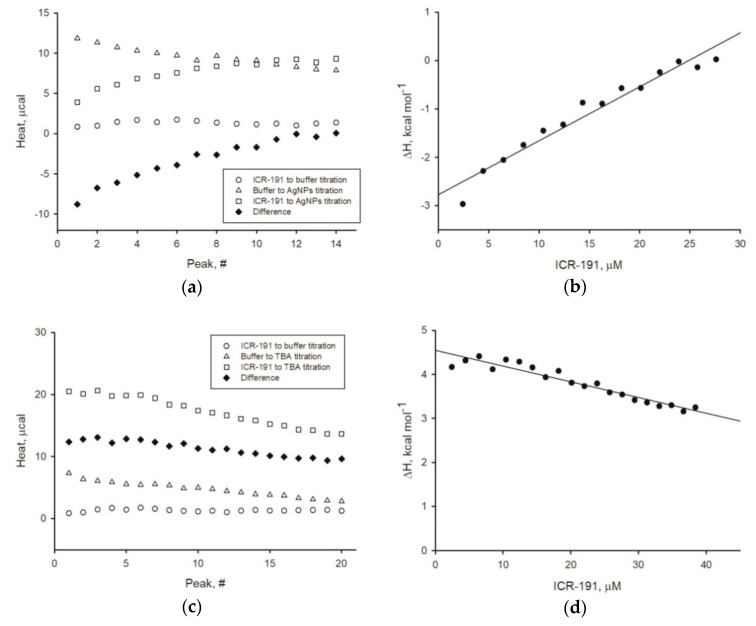

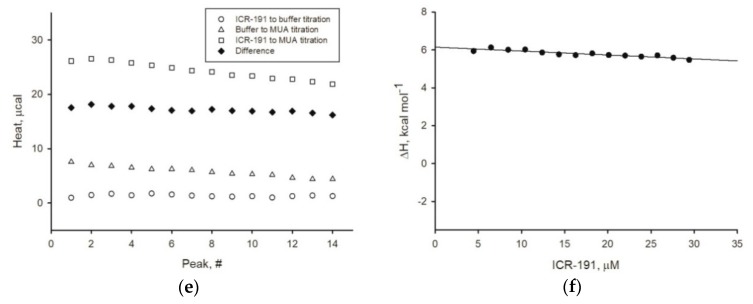
Thermodynamical properties of AgNPs and ICR-191 interactions. (**a**) Thermal effects of titrations of naked AgNPs with ICR-191 (squares), buffer with ICR-191 (circles), and naked AgNPs with buffer (triangles). Black diamonds represent difference between the heat of naked AgNPs titration with ICR-191 and sum of heats obtained for control titrations (buffer-ICR-191, naked AgNPs-buffer); (**b**) heat of ICR-191-naked AgNPs interactions (corrected for background thermal effects as described above), calculated as kcal mol^−1^ of injected ICR-191. The enthalpy change (ΔH) of ICR-191-naked AgNPs interactions, calculated by the linear regression (R^2^ = 0.952) of experimental points to [ICR-191] tending to zero, is equal to −2.775 ± 0.123 (±SE) kcal mol^−1^; (**c**) thermal effects of titrations of AgNP-TBA with ICR-191 (squares), buffer with ICR-191 (circles), and AgNP-TBA with buffer (triangles). Black diamonds represent difference between the heat of AgNP-TBA titration with ICR-191 and sum of heats obtained for control titrations (buffer-ICR-191, AgNP-TBA-buffer); (**d**) heat of ICR-191-AgNP-TBA interactions (corrected for background thermal effects as described above), calculated as kcal mol^−1^ of injected ICR-191. The enthalpy change (ΔH) of ICR-191-AgNP-TBA interactions, calculated by the linear regression (R^2^ = 0.918) of experimental points to [ICR-191] tending to zero, is equal to 4.547 ± 0.059 (±SE) kcal mol^−1^; (**e**) thermal effects of titrations of AgNP-MUA with ICR-191 (squares), buffer with ICR-191 (circles), and AgNP-MUA with buffer (triangles). Black diamonds represent difference between the heat of AgNP-MUA titration with ICR-191 and sum of heats obtained for control titrations (buffer-ICR-191, AgNP-MUA-buffer); (**f**) heat of ICR-191-AgNP-MUA interactions (corrected for background thermal effects as described above), calculated as kcal mol^−1^ of injected ICR-191. The enthalpy change (ΔH) of ICR-191-AgNP-MUA interactions, calculated by the linear regression (R^2^ = 0.839) of experimental points to [ICR-191] tending to zero, is equal to 6.147 ± 0.049 (±SE) kcal mol^−1^.

We also performed precise thermodynamical analysis to exclude ambiguities from previous experiments. Using the ITC technique, we were able to follow the heat changes resulting from possible interactions between AgNPs and ICR-191. Figure 7 presents thermograms of ICR-191 titrated with buffer, buffer with tested AgNPs, and ICR-191 titrated with nanostructures. The final thermal effect of AgNPs–ICR-191 interactions was evaluated after considering heats of dilution processes of every component in the experiments (Figure 7a,c,e). We subtracted the heats measured for control samples from the heat of analyzed AgNPs titrated with ICR-191, and the obtained corrected heat is presented on plots (Figure 7b,d,f). Finally, we determined enthalpy changes values for three types of AgNPs: for naked AgNPs-ICR-191 interactions ΔH = −2.775±0.123 (±SE) kcal mol^−1^; for AgNP-TBA–ICR-191 interactions ΔH = 4.547 ± 0.059 (±SE) kcal mol^−1^;and for AgNP-MUA–ICR-191 interactions ΔH = 6.147 ± 0.049 (±SE) kcal mol^−1^. Considering obtained data, we hypothesize that only naked AgNPs formed heteroaggregates with ICR-191 as a result of the spontaneous, exothermic reaction.

### 3.4. Biological Activity of Silver Nanoparticles

**Figure 8:**
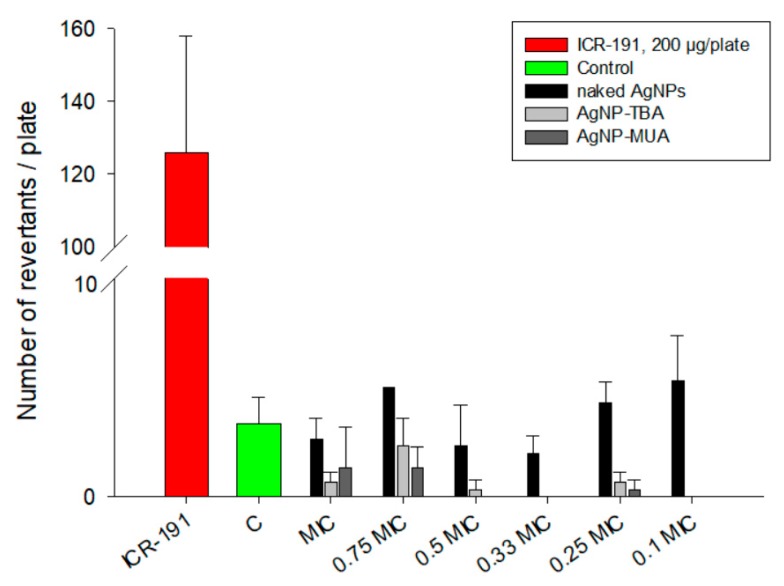
Mutagenic activity of different AgNPs in *S. typhimurium* TA98 mutagenicity test: 200 µg/plate of ICR-191 was used as a positive control (red column). C—negative control, bacteria incubated with water. Purple columns represent bacteria incubated with naked AgNPs in a 0.09–0.9 µg/mL concentration range. Light blue columns represent bacteria incubated with AgNP-TBA in a 0.078–0.78 µg/mL concentration range. Navy blue columns represent bacteria incubated with AgNP-MUA in a 0.066–0.66 µg/mL concentration range. Results are reported as mean number of revertants ±SD. MIC—minimum inhibitory concentration.

To verify the influence of different coating agents on AgNPs’ mutagenic activity, we applied bacterial reverse mutation assay, called the Ames test. The obtained results presented in Figure 8 indicate that tested AgNPs showed no mutagenicity toward *S. typhimurium* TA98. For the broad ranges of naked AgNPs, AgNP-TBA, and AgNP-MUA concentrations, the observed effects were similar to the negative control response. Model acridine mutagen ICR-191, chosen as a positive control, induced the expected mutagenic potential. It is worth it to emphasize that the AgNPs concentrations used in this assay were not lethal towards *S. typhimurium* TA98. Their concentrations ranges (0.09–0.9 µg/mL for naked AgNPs, 0.078–0.78 µg/mL for AgNP-TBA, and 0.066–0.66 µg/mL for AgNP-MUA) were selected in accordance to previously determined minimum inhibitory concentrations (MIC) and minimum bactericidal concentrations (MBC) for tested bacteria. 

Ultimately, we analyzed the toxicity of naked AgNPs and TBA- and MUA-coated AgNPs on HaCaT and MelJuSo cell lines. These biological models were chosen to assess potential differences in nanoparticles effects on cells viability on both non-cancerous and cancerous cells. Cells viability was tested with AlamarBlue assay. In this test, AlamarBlue reagent is converted into red color dye by healthy and living cells, which can be quantified by absorbance measurements. Our results showed that each among the three types of AgNPs had a mild toxic effect on both tested cell lines (data not shown). The cytotoxic effects were proportional to silver concentration, thus the highest effect was observed after naked AgNPs’ treatment and the lowest after incubation with AgNP-MUA. Additionally, tested AgNPs did not change significantly the ICR-191 activity against HaCaT and MelJuSo cell lines. Only in the case of AgNP-MUA treatment, a minor enhancement in the acridine mutagen’s cytotoxic effect against the tested cells was observed.

## 4. Discussion

Nowadays, advances in the development of nanotechnology have raised concerns over the influence of a broad range of nanoparticles on human health and the environment. However, despite extensive studies, knowledge about the safety of AgNPs usage, especially when their surface is modified, is relatively poor and several questions still remain to be answered. On the other hand, evolving techniques of nanoparticles surface functionalization seem to be a tool for more efficient control of the nanoparticles action and, therefore, their more beneficial use in nanomedicine. For example, when using silver as a wound healing agent, pertinent selection of substituents could enhance the antimicrobial effect of AgNPs, while limiting their toxic effects on the skin cells. Here, we aim to describe the possible changes in nanosilver physical and biological properties, induced by surface coatings. 

The first step of our study was the AgNPs’ synthesis, based on the new methodology developed by our group [17], and their further surface functionalization. Thiobarbituric acid (TBA) and 11-mercaptoundecanoic acid residues (MUA) were attached to newly synthesized AgNPs. The synthesis efficiency was defined as the content of silver atoms in each of the three samples: naked AgNPs, AgNPs coated with TBA, and AgNPs coated with MUA. On the basis of literature reports, TBA is adsorbed on AgNPs through a surface chelation involving S and N atoms. Previous studies also demonstrated that TBA provides stabilization of nanostructures and better aqueous dispersibility [21]. Botasini et al. [22] showed that TBA not only stabilizes AgNPs structures, but also allows for some non-spherical shape modifications. Moreover, studies suggested that after TBA adsorption on AgNPs’ surface, TBA’s functional groups might interact with several proteins residues [21]. Conversely, MUA is likely to adsorb to AgNPs’ surface through the thiol (-SH) or the carboxylate group (−COO−) [23]. AgNPs that are coated with MUA residues could thus become more hydrophilic than naked AgNPs [24]. 

Precise data about particles size, shape, and morphology are essential for a full evaluation of synthesized nanomaterials. To provide the required pieces of information, the use of more than one analytical method is recommended. Therefore, in our publication, naked AgNPs, AgNP-TBA, and AgNP-MUA nanoparticles were evaluated with a broad range of biophysical methods, such as SEM, AFM, DLS, and UV/vis spectroscopy.

In our research, we used two different microscopy technologies. SEM is a direct approach indicating size range and shape of synthesized AgNPs. Thanks to this approach, we visualized three types of AgNPs as the bright spots in the center of salt crystals (residues after synthesis, crystallized after the evaporation of water). Naked AgNPs nanoparticles, as well as nanostructures coated with TBA and MUA ligands, resemble a square shape. In our case, the introduction of surface modifications did not change the shape of the nanoparticles [15,20,22]. The AFM technique provided an additional insight into the AgNPs’ morphology and height. Naked AgNPs, AgNP-TBA, and AgNP-MUA suspensions can be characterized by a high degree of dispersion. It also should be noted that the layer of polyvinylpyrrolidone polymer, which has not been fully removed after the synthesis process, can be observed on the surface of each of the visualized AgNPs, evidenced by the blurring of their images and height alterations. We assume that the polymer film smoothed the surface of the nanostructures, masking their possible sharp edges. As a result, all AgNPs appear in the AFM pictures as sphere-like objects, irrespective of their real geometrical shape. 

The second approach involved the investigation of naked AgNPs, AgNP-TBA, and AgNP-MUA aggregates’ size distribution profile with DLS, combined with electrokinetic potential (ζ potential) determination. Clearly, the addition of TBA and MUA ligands to naked AgNPs surface resulted in nanoparticles’ hydrodynamic diameter size enlargement. This change might be associated with TBA’s ability to influence nanoparticle size and solute–water attractive interactions on hydration water structure around hydrophobic solutes [22]. In addition, the presence of TBA can promote nanoparticles’ homoaggregation, which might affect nanoparticles’ bioavailability. Similarly, AgNP-MUA nanoparticles also aggregate, which might explain our observations [24]. Nevertheless, aggregation does not necessarily correlate with toxicity. This hypothesis might be of interest for AgNP-TBA’s and AgNP-MUA’s future biological application, because smaller nanoparticles, rather than bigger ones, might have enhanced toxicity [9,22,25]. Electrokinetic potential is proposed as a standard analytical measurement for nanoparticles’ surface characterization, and can provide insight into nanoparticles’ ability to interact with other macromolecules (like proteins) or to penetrate through cells membranes. However, it is worth mentioning that the ζ potential value depends on variable experimental conditions, such as temperature, pH, ionic strength of solvent, and solvent viscosity. Changes in ζ potential can be attributed to changes in the amount of charged ions at the nanoparticles’ surfaces [4,6,11]. The naked AgNPs nanoparticles have the most positive average surface charge (−0.65 mV), compared with nanoparticles modified with TBA and MUA coatings. We can hypothesize that the surface coating decreased the electrokinetic potential of the tested nanostructures. The negative average surface charges can also be triggered by AgNPs’ high tendency to aggregate [4,26,27]. AgNPs described in the literature had negative ζ potential values too [3,4,11,26]. The authors of cited publications presented a wide range of ζ potential values reaching from −0.9 mV to even −46 mV. By comparing these results together, we can conclude that ζ potential alone is not a sufficient factor for the AgNPs’ stability analysis. AgNPs can also be sterically stabilized by organic surface coatings, independent of surface charge. Moreover, we should also note that nanoparticles’ shielding with the polyvinylpyrrolidone leftovers can reduce the negative charge. In our opinion, the presented reduction of ζ potential values in 10-fold diluted samples to the level of approximately 13 mV can be the effect of AgNPs deaggregation and their lower tendency to form homo-aggregates, which stands in agreement with the literature [3,4,27].

Further naked AgNPs, AgNP-TBA, and AgNP-MUA characterization and their stock suspensions stability were monitored by UV/vis spectroscopy. AgNPs, in general, are characterized by a strong optical density, due to plasmon resonance [4]. Many reports suggest that the band located in the region of approximately 420 nm corresponds to the surface plasmon resonance of silver [2,12,28]. However, only AgNP-MUA suspension had a clear absorbance peak at 405 nm. Moreover, all three types of AgNPs strongly scatter the light, which might be because of their homoaggregation in the suspension buffer. 

In recent years, the use of metallic and non-metallic nanoparticles as drug carriers has risen concern [2,9,15,29,30,31]. Therefore, to take full advantage of AgNPs’ potential and use them as a potential treating agent or delivery platform in a controlled way, it is necessary to precisely understand their synthesis, characterization, as well as biological interactions (transport, uptake, and further degradation in cells and organisms). The investigation of potential interactions between nanostructures and biologically active substances is also crucial. Siddiqi et al. reported that the presence of Ag in drug delivery systems improves medicines absorption into treated cells [28]. In our study, we investigated possible heteroaggregation between model acridine mutagen ICR-191 molecules and three types of AgNPs. These interactions resulted in variations of biophysical properties of nanoparticles. 

Spectroscopic analysis of potential interactions of naked AgNPs, AgNP-TBA, or AgNP-MUA with ICR-191 required two independent titrations, to eliminate the effect of light scattering by tested AgNPs. On the basis of obtained spectra indicating slight batho- and hypochromic shifts, we assume the existence of interactions between model acridine mutagen and naked AgNPs or AgNPs coated with the aliphatic MUA. Thus, it can be speculated that TBA on a surface of AgNPs, as a heterocyclic compound with two nitrogen heteroatoms in its structure, probably could prevent similar interactions with the aromatic ICR-191. Moreover, changes in absorbance maxima positions might be correlated with heteroaggregates’ formation in tested samples [15,25].

As the performed analysis did not allow us to make more explicit conclusions, and in order to understand possible interactions between AgNPs with ICR-191 in a more detailed way, we applied fluorescence spectroscopy. If ICR-191 molecules were involved in heteroaggregates with naked AgNPs, AgNP-TBA, or AgNP-MUA nanostructures, this would result in changes of the intensity of fluorescence light emitted by the acridine mutagen. This decrease was observed when the fluorescence intensity maximum at 496 nm was altered in the experiment in which ICR-191 was titrated with naked AgNPs. This indicates that naked AgNPs directly interacted with mutagen molecules. The lack of fluorescence changes after AgNP-TBA and AgNP-MUA addition to ICR-191 molecules might be explained by a reduced interaction after nanoparticles’ surface modification. Introduction of TBA and MUA residues resulted in nanoparticles’ size enlargement and homoaggregation enhancement. Hence, their availability for ICR-191 interaction could be limited [3,8,24,32,33]. 

In an additional DLS analysis, the hydrodynamic diameter values determined for AgNP-MUA nanoparticles alone were equal to 38.9 and 189.8 nm. After ICR-191 addition to the tested mixture, there was a visible increase in the hydrodynamic radius size (73.8 and 308.0 nm), which might be interpreted as a formation of AgNP-MUA-ICR-191 heteroaggregates. Such extensions of the hydrodynamic diameter were previously described in the analysis evaluating the heteroaggregation of C_60_ fullerene with ICR-191 [15], and with the anticancer drugs doxorubicin [34] and cisplatin [35]. In the case of other types of silver nanostructures, no changes in aggregate sizes were observed. These differences probably again can be explained by the different surface functionalization of tested AgNPs. AgNPs coated with MUA, the thiol-containing fatty acid used in the design of monolayers and metal-associated nanoparticles, become more hydrophilic, which might facilitate the mixed-aggregates formation with acridine mutagen molecules. 

Furthermore, to dispel any doubts arising from previous experiments, we determined the enthalpy change values for naked AgNPs, AgNP-TBA, or AgNP-MUA titrated with ICR-191. The enthalpy change values (ΔH) calculated for AgNPs after surface modifications were above zero kcal mol^-1^, which suggests the absence of potential interactions. On the basis of the obtained results, we can state that only naked AgNPs directly interacted with ICR-191 molecules as a result of the spontaneous exothermic reaction (ΔH equals −2.775 kcal mol^−1^). For a comparison, a similar negative enthalpy change (ΔH = −8.48 kcal mol^−1^) was observed in a study analyzing the direct interaction of ICR-191 with C _60_ fullerene [15]. Loran et al. [36] suggest that zerovalent AgNPs’ surface may form covalent bonds with several atmospheric and solution components. As MUA and TBA residues had already interacted with zerovalent silver at nanoparticles surface, coated nanoparticles could not bind ICR-191, because there was no remnant zerovalent silver and MUA and TBA residues did not interact with ICR-191.

The Ames test is universally used to examine the mutagenic activity of different compounds. This assay, based on *S. typhimurium* TA 98 incubation in the presence of naked AgNPs, AgNP-TBA, or AgNP-MUA, is able to detect eventual DNA damages, like point mutation or small deletions, induced by nanostructures. Experiments were conducted in an environment that is non-toxic to bacteria, employing wide range of nanoparticles concentrations, which were previously selected on the basis of MIC/MBC evaluation (data not shown). As expected, the naked AgNPs, AgNP-TBA, and AgNP-MUA did not exhibit mutagenicity at tested concentrations, compared with ICR-191 with well-known mutagenic potential. Data published by Butler et al. [3], Doak et al. [37], and Li et al. [25] performed with several bacterial strains (*S. typhimurium* TA98, TA100, and TA102) are in agreement with our results. In their studies, AgNPs also did not exhibit mutagenic potential. In contrast, there are a few publications describing some bacterial genotoxic changes triggered by AgNPs. Stensberg et al. [4] suggested that AgNPs can be more toxic to prokaryotes than Ag^+^. It is worth underlining that mentioned tests examine different types of DNA alterations: micronucleus assay indicates fragmental chromosomal damage and comet assay measures the existence of DNA strands breaks [3,37]. The mentioned studies suggest that observed positive results in genotoxic assays can be attributed to AgNPs-related oxidative stress. On the other hand, a lack of genotoxicity might be related not only to a lack of AgNPs mutagenic potential, but also to a decreased transport of nanoparticles to the cell interior, which may be because of the particles’ larger sizes, average surface charges, or surface modifications [5,6,25,38]. Another possible explanation for the negative results observed for AgNPs in the bacterial reverse mutation test is that naked AgNPs, AgNP-TBA, and AgNP-MUA may act via genotoxic mechanisms, which cannot be visualized using bacterial assays.

Finally, a possible impact of AgNPs surface functionalization on cells toxicity was assessed in eukaryotic cells, namely the non-cancerous HaCaT and the cancerous MelJuSo cell lines. Nanoparticles cytotoxicity was assessed with AlamarBlue cytotoxicity assay. We also checked if naked AgNPs, AgNP-TBA, and AgNP-MUA might modulate model acridine mutagen activity, which is known for its toxicity. Regardless of the incubation time and cell type, each of the tested AgNPs triggered a minor decrease in cells viability, compared with the negative control—not treated cells. These toxic effects may be related to the amount of silver contained in different nanostructures, because the level of naked AgNPs toxicity was higher than that observed in cells after AgNP-MUA treatment (data not shown). The higher silver concentration and the direct interaction of cells’ proteins with AgNPs surfaces may induce a greater release of Ag^+^ ions, which are considered responsible for nanoparticles toxicity. In AgNP-TBA and AgNP-MUA nanoparticles, oxidation and subsequent Ag^+^ release can also be limited, because of their changed surface chemistry [2,3,8,10]. In the previous research conducted by our group, we repeatedly observed a modulating mechanism of different nanoparticles against tested mutagens and anticancer drugs [15,29,34,39,40]. Surprisingly, the naked AgNPs, AgNP-TBA, and AgNP-MUA, in general, do not modulate acridine mutagen activity. Only in the case of cells treated with a mixture of ICR-191 and AgNP-MUA, a slight increase in toxicity was apparent (data not shown). The decrease in cells viability might be connected with a synergistic effect of ICR-191 and AgNP-MUA nanoparticles. AgNP-MUA has a described tendency to aggregate, which might lead to the increased toxicity, explained by clouding of the cell membranes, and thereby facilitates ICR-191 activity [23,24,41]. Many cells lines that interact with AgNPs have been cultured and studied so far, including red blood cells, L929 fibroblasts, RAW 264.7 murine macrophages, L5178Y lymphoma cells, BRL3A rat liver cells, PC-12 pheochromocytoma-derived cell neuroendocrine cells, GSCs germ line stem cells, RBE4 rat brain endothelial cells, MCF-7 human breast adenocarcinoma cells, HepG2 human liver cells, BEAS-2B human bronchial epithelial cells, A4549 lung alveolar epithelial cells, and hMSC (human mesenchymal stem cells). Yet, according to the contradicting information available in the published literature, the toxicity and IC_50_ values of different AgNPs vary, probably depending on tested cell types and nanoparticles’ size, shape, and surface modification [2,3,4,5,7,10,11,42]. The “Trojan horse” mechanism, proposed by several scientists, seems like another interesting argument in the discussion concerning AgNPs biological activity [8,43]. According to this theory, AgNPs serve as transporting platforms, which can penetrate cells membranes and release a high amount of toxic Ag^+^ ions. The release of Ag^+^ ions is a pH-dependent process, which might be useful for future selective cancer treatments, based on the pH difference in healthy and cancerous cells. Interestingly, some of the components of cell culture medium, like fetal bovine serum, can induce much larger silver ion release rates when compared with water [44]. Reports also suggest that determination of the free silver ions concentration in the intracellular compartment might also be used to address this complex toxicity phenomenon, which still needs to be elucidated [2,4,43]. Concluding pieces of information mentioned above with our results, we believe that it is extremely difficult to unambiguously determine the toxic potential for all kinds of AgNPs.

## 5. Conclusions

Our article presents AgNPs’ surface modification protocols, which allow obtaining nanoparticles that interact differently between themselves and the adjacent (bio)molecules. We thus efficiently synthesized AgNPs coated with TBA and MUA residues and described their physicochemical characterization. Subsequently, we evaluated the effect of ligands’ introduction on the biological activity of analyzed nanoparticles using a wide range of biophysical methods. We observed that the modifications in AgNPs surfaces affect nanoparticles’ ability to self-aggregate, as well as to interact with small molecules by forming heteroaggregates. AgNPs coated with MUA showed the most interesting interactions with ICR-191, slightly modulating its toxicity properties by decreasing the viability in treated cells. The nature of these interactions is probably complex and can be attributed to different interactions of nanoparticles with the surrounding environment. While the nature of such interactions was not precisely assessed in our study, we could have a preliminary insight into how such interactions affect different nanoparticles’ biophysical characterization procedures. Further studies are thus necessary to assess the biological activity of both AgNPs and small molecules involved in the mentioned interactions. As confirmed by our study, AgNPs’ surface functionalization is a powerful tool, which can govern nanostructures’ interactions with biological systems, and efforts should be undertaken to address surface coating-dependent toxicity.

## Figures and Tables

**Table 1:** Concentrations of Ag (µg/mL) in samples *. AgNPs—silver nanoparticles; TBA—thiobarbituric acid; MUA—11-mercaptoundecanoic acid.

Sample	Concentration, µg/mL	RSD, %
naked AgNPs	2.65 × 10^3^	1.6
AgNP-TBA	625	2.2
AgNP-MUA	65.5	0.75

* Average values (n = 3); RSD—relative standard deviation.

**Table 2:** Hydrodynamic diameter, polydispersity index (PDI), and ζ potential value measured for synthesized AgNPs *.

Sample	Hydrodynamic Diameter, nm	PDI	ζ Potential, mVin Original Samples	ζ Potential, mVin 10-Fold Diluted Samples
naked AgNPs	110 ± 4	0.22 ± 0.01	−0.65 ± 0.03	−12.5 ± 0.5
AgNP-TBA	273 ± 3	0.29 ± 0.01	−7.92 ± 0.31	−13.3 ± 0.6
AgNP-MUA	199 ± 2	0.24 ± 0.01	−4.89 ± 0.20	−13.1 ± 0.6

* Dynamic light scattering (DLS) measurements were performed in triplicate. Results are presented as mean values ± SD.
